# Supplementary material for: Antisense transcription‐dependent chromatin signature modulates sense transcript dynamics
Source: Mol Syst Biol. 2018 Feb 12;14(2):e8007. doi: 10.15252/msb.20178007 (PMC5810148; doi:10.15252/msb.20178007)
Supplement: Supplementary file 1 — Appendix [file MSB-14-e8007-s001.pdf]

Appendix: Antisense transcription-dependent chromatin signature modulates sense transcript dynamics

Thomas Brown*, Françoise S. Howe*, Struan C. Murray*, Meredith Wouters, Philipp Lorenz, Emily Seward, Scott Rata, Andrew Angel[§] and Jane Mellor[§]
Department of Biochemistry, South Parks Road, Oxford, OX1 3QU, UK.

Contents:

Appendix Figure S1

Supplementary Tables

- **Appendix Table S1. Primer sequences used for qPCR.**
- **Appendix Table S2. Primer sequences used to generate the strand-specific Northern blotting probes**
- **Appendix Table S3. Sequences of the *GAL1* sense RNA-FISH probes**
- **Appendix Table S4. Reagents**
- **Appendix Table S5. Software and Algorithms**
- **Appendix Table S6. Organisms/Strains**

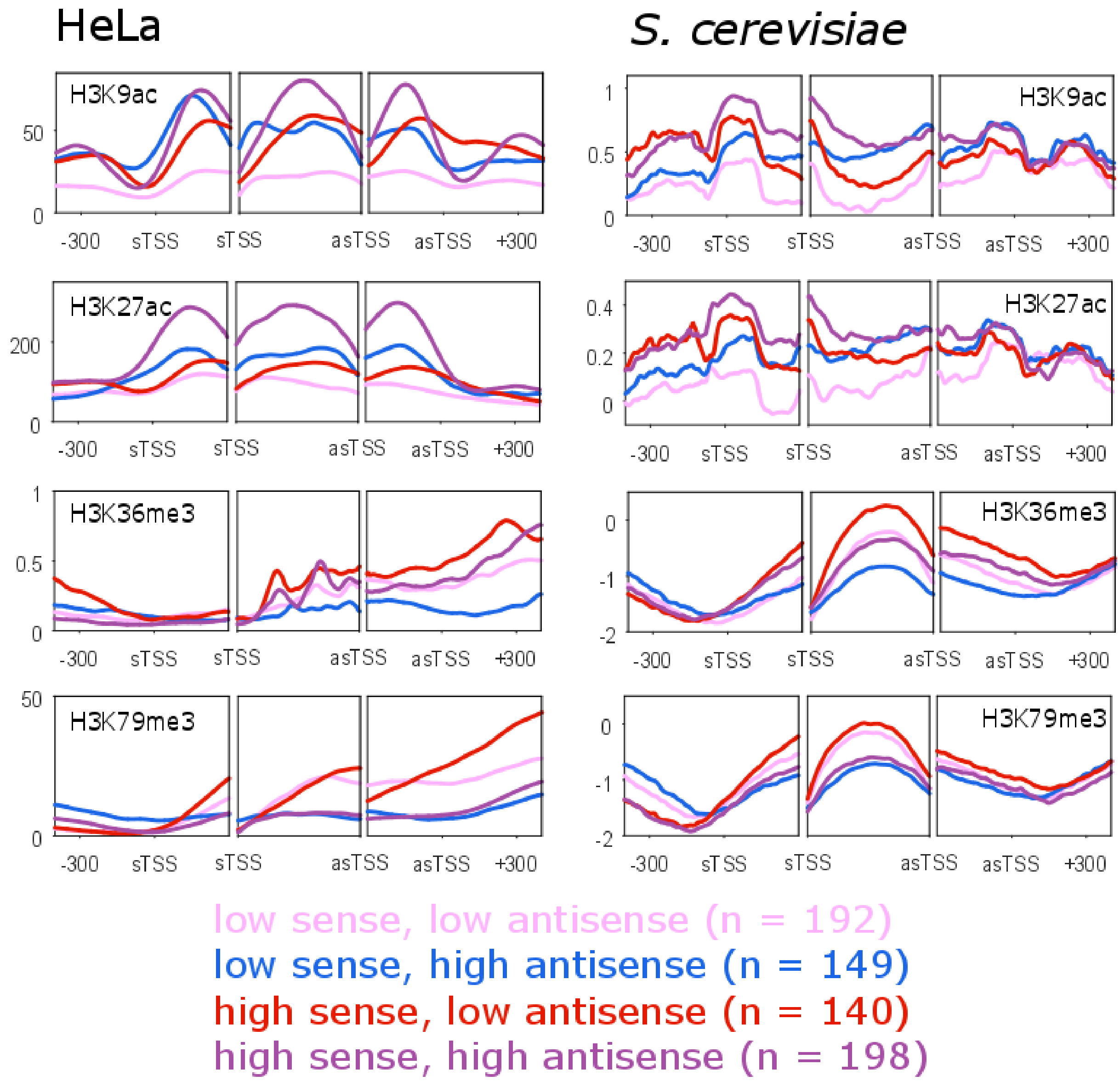

Appendix Figure S1

Levels of histone acetylation and trimethylation in gene classes with varying levels of antisense transcription, in both HeLa and *S. cerevisiae*. Shown are average levels in four different gene classes as shown at bottom of figure. In all cases, *low* was any level of sense/antisense transcription (measured by NET-seq) beneath the 30th percentile for all genes, and *high* was any level above the 70th percentile. For each modification in each species, the three panels are the same format as shown in fig 2B.

Supplementary Tables

Appendix Table S1. Primer sequences used for qPCR.

**Primer Name**	**Sequence (5'-3')**
GAL1 -2 F	CCGGTCGCGTTCCTGAAACG
GAL1 -2 R	GCCAGGTTACTGCCAATTTT
GAL1 -1 F	TGGAAAAGCTGCATAACCACT
GAL1 -1 R	GCGCTAGAATTGAACTCAGGT
GAL1 1 F	CGCTTATGATGCTAAACCGG
GAL1 1 R	CGCAAAGCATATCAAAATCA
GAL1 2 F	CCGTCAAAGTTTTGAACGAGA
GAL1 2 R	CAGAAGGATCAATTGTGACATAAG

Appendix Table S2. Primer sequences used to generate the strand-specific Northern blotting probes

**Primer Name**	**Sequence (5'-3')**
GAL1 F	TGTGTCGGACTGGTCTAATT
GAL1 R	GATCCATACCGCCATTGTTA

Appendix Table S3. Sequences of the *GAL1* sense RNA-FISH probes

Underlined nucleotides were ordered as amino-allyl-dTs for subsequent labelling with Cy3 fluorescent dye.

**Probe Name**	**Sequence (5'-3')**
GAL1 Sense probe 1	T**T**ATGCTCGGGCAC**T**TTTCGGCCAA**T**GG<br>TCTTGGTAA**T**TCCTTTGCGCT
GAL1 Sense probe 2	AGGA**T**CAATTGTGACATAAGAACCG**T**CC<br>AACGGCAAA**T**CGAACTTCC**T**T
GAL1 Sense probe 3	**T**ACATCACCC**T**CACAGAAGACT**T**GCAGC<br>CCGGCCAGAGGAGCAC**T**GGCAAA
GAL1 Sense probe 4	**T**CCAGGGCCCATA**T**TCGCTTTAACAACA<br>GC**T**AAAGCAACGGCACAAA**T**G
S65T pFA6a-GFP probe 1	CCTCTTCTTGAAAAGTGACC
S65T pFA6a-GFP probe 2	ATCTACCACTACAATTACCC

S65T pFA6a-GFP probe 3	TAAAAGACAGTCACCTCTCC
S65T pFA6a-GFP probe 4	CGTTGTATGCCTTTTGAATG
S65T pFA6a-GFP probe 5	AACGTGATGACCTTTTGATG
S65T pFA6a-GFP probe 6	CAAGGTACCGGTTGTGAACA
S65T pFA6a-GFP probe 7	AGTGAATACCACAAGTTACG
S65T pFA6a-GFP probe 8	CTAGTATACTTTGCCGTACT
S65T pFA6a-GFP probe 9	AAAAGTTCTCACGGTACGGG
S65T pFA6a-GFP probe 10	TCCAATACATGTCCTTTCTT
S65T pFA6a-GFP probe 11	AAAAGTTTCTACTGCCCTTG
S65T pFA6a-GFP probe 12	GTTCTGTGCACGACCTCAGT
S65T PFA6A-GFP probe 13	ACTTCCACTATGGGAACAAT
S65T PFA6A-GFP probe 14	TCTTAGCTCAATTTTCCATA
S65T pFA6a-GFP probe 15	TACCTTTGTAAGAACCTGTG
S65T pFA6a-GFP probe 16	CCTTATGTTGATATTGAGTG
S65T pFA6a-GFP probe 17	TACATATGTAGTACCGTCTG
S65T pFA6a-GFP probe 18	TGTTTTCTTACCTTAGTTTC
S65T pFA6a-GFP probe 19	TCTGTGTTGTAACTTCTACC
S65T pFA6a-GFP probe 20	CGCAAGTTGATCGTCTGGTA
S65T pFA6a-GFP probe 21	TGTTTTATGAGGTTAACCGC
S65T pFA6a-GFP probe 22	CGGGACAGGAAAATGGTCTG
S65T pFA6a-GFP probe 23	ACAGGTGTGTTAGACGGGAA
S65T pFA6a-GFP probe 24	CTTTCTAGGGTTGCTTTTCT
S65T pFA6a-GFP probe 25	CTGGTGTACCAGGAAGAACT
S65T pFA6a-GFP probe 26	AACATTGTCGACGACCCTAA
S65T pFA6a-GFP probe 27	GTACCGTACCTACTTGATAT

Appendix Table S4. Reagents

REAGENT or RESOURCE	SOURCE	IDENTIFIER
**Antibodies**		
Pan-H3 C terminus	Millipore	Cat# 07-690<br>RRID:AB_417398

H3K4me2	Millipore	Cat# 07-030 RRID:AB_11213050
H3K4me3	Millipore	Cat# 05-745R RRID:AB_1587134
H3K9ac	Millipore	Cat# 07-352 RRID:AB_310544
**Chemicals, Peptides, and Recombinant Proteins**		
SYBR green qPCR master mix	Bioline	Cat# QT605-20
CyDye™ Cy3	GE Healthcare	Cat# PA23001
Protein A Sepharose CL4B beads	VWR	Cat# 17-0780-01
**Critical Commercial Assays**		
QIAquick PCR Purification Kit	Qiagen	Cat# 28106
QIAquick Nucleotide Removal Kit	Qiagen	Cat# 28304
**Oligonucleotides**		
For Northern blotting probe primers see Table S2		
For qPCR primers see Table S1		
For RNA FISH probes see Table S3		

Appendix Table S5. Software and Algorithms

**Software and Algorithms**		
Fiji/ImageJ	*(Schindelin et al., 2012)*	https://imagej.net/Fiji
MATLAB, including Image Processing and Statistics and Machine Learning Toolboxes	*The Mathworks, Inc.*	
Analysis of Northern blotting and decay rates in MATLAB: display_mRNAShutdown.m	This paper	

Gillespie algorithm of RNA production and degradation written in C/CUDA: calculate_metrics.h, cuda_gillespieReduced.h, cuda_randomFuncs.h, file_funcs.h, sample_params.h, cuda_paramSearchSeparate.cu, degradation_confidenceIntervals.m	This paper	
Analysis of FISH images and quantification of RNA in Matlab: FindAndAnalyseFoci.m, DetermineCutoffs.m, DetectCellsAndQuantifyFoci.m	This paper	
MIJI		https://imagej.net/Miji
CellProfiler, IdetifySecPropagateSubFunction (Matlab compiled function from Cellprofiler 1.0)	(Carpenter et al., 2006)	http://www.cellprofiler.org
Piotr's Matlab Toolbox, nonMaxSupr function		https://pdollar.github.io/toolbox/

Appendix Table S6. Organisms/Strains

**Experimental Models: Organisms/Strains**	
S. cerevisiae: Name = BY4741; Genotype = MATa; *his3*Δ1; *leu2*Δ0; *met15*Δ0; *ura3*Δ0	Euroscarf
S. cerevisiae: Name = *GAL1:ADH1*t (high antisense); Genotype = *GAL1:ADH1*t:HISMX	(Murray et al., 2012)
S. cerevisiae: Name = TATA mutant (low antisense); Genotype = *GAL1:ADH1*t (scramble):KANMX	(Murray et al., 2015)
S. cerevisiae: Name = *GAL1:ADH1*t (high antisense) *set3Δ*; Genotype = *GAL1:ADH1*t:HISMX *SET3::*KANMX	This study

S. cerevisiae: Name = TATA mutant (low antisense) *set3Δ*; Genotype = *GAL1:ADH1*t (scramble):KANMX *SET3::*HISMX	This study
S. cerevisiae: Name = BY ATP4-GFP; Genotype = *ATP4:GFP*:HISMX	This study
S. cerevisiae: Name = BY GCV3-GFP; Genotype =*GCV3:GFP*:HISMX	This study
S. cerevisiae: Name = BY HMS2-GFP; Genotype =*HMS2:GFP*:HISMX	This study
S. cerevisiae: Name = BY TIM17-GFP; Genotype =*TIM17:GFP*:HISMX	This study
S. cerevisiae: Name = BY URA4-GFP; Genotype =*URA4:GFP*:HISMX	This study
