# Supplementary material for: Antisense transcription‐dependent chromatin signature modulates sense transcript dynamics
Source: Mol Syst Biol. 2018 Feb 12;14(2):e8007. doi: 10.15252/msb.20178007 (PMC5810148; doi:10.15252/msb.20178007)
Supplement: Supplementary file 2 — Expanded View Figures PDF [file MSB-14-e8007-s002.pdf]

Expanded View Figures

Figure EV1. Sense and antisense transcription have similar relationships in yeast and humans.

A The average levels of sense transcription determined for both HeLa and *Saccharomyces cerevisiae* using NET-seq. For each trio of panels, the left panel shows average levels around the sTSS, the right panel shows the average levels around the asTSS, and the middle panel shows the average level within thirty equal sized bins within the region bound by the sTSS and asTSS. The top panels compare sense transcription levels between two sets of genes—those with high levels of sense transcription (dark red), and those with low levels (pale red), determined by GRO-seq in HeLa cells and PRO-seq in yeast. The bottom panels show those genes with high levels of *antisense* transcription (dark blue), and those with low levels (pale blue).

B The average levels of antisense transcription determined for both HeLa and *S. cerevisiae* using NET-seq, with panels arranged as in (A).

C, D The average levels of nucleosome occupancy determined for both (C) HeLa and (D) *S. cerevisiae* using MNase-seq. For each trio of panels, the left panel shows average levels around the sTSS, the right panel shows the average levels around the asTSS, and the middle panel shows the average level within thirty equal sized bins within the region bound by the sTSS and asTSS. The top panels compare two sets of genes—those with high levels of sense transcription (dark red), and those with low levels (pale red), determined by GRO-seq in HeLa cells and PRO-seq in yeast. The bottom panels show those genes with high levels of *antisense* transcription (dark blue), and those with low levels (pale blue).

E Distributions of sense transcription reads for genes in HeLa cells and *S. cerevisiae*. Average values per base pair were calculated within the first exon of HeLa cells, and the whole gene of *S. cerevisiae*. Two gene groups were compared—those with an asTSS (dark blue) and those without (light blue). The *P*-value was determined using the Wilcoxon rank sum test.

F, G Scatter plots comparing the number of GRO-seq (HeLa) or PRO-seq (*S. cerevisiae*) reads at two different windows and orientations, as shown in the gene diagrams. (F) Levels of antisense transcription were compared to downstream sense transcription in both HeLa cells and *S. cerevisiae*. (G) Levels of sense transcription were compared to upstream divergent transcription in both HeLa cells and *S. cerevisiae*. Shown for in all cases is the Spearman correlation coefficient, r_s.

 © 2018 The Authors

 © 2018 The Authors

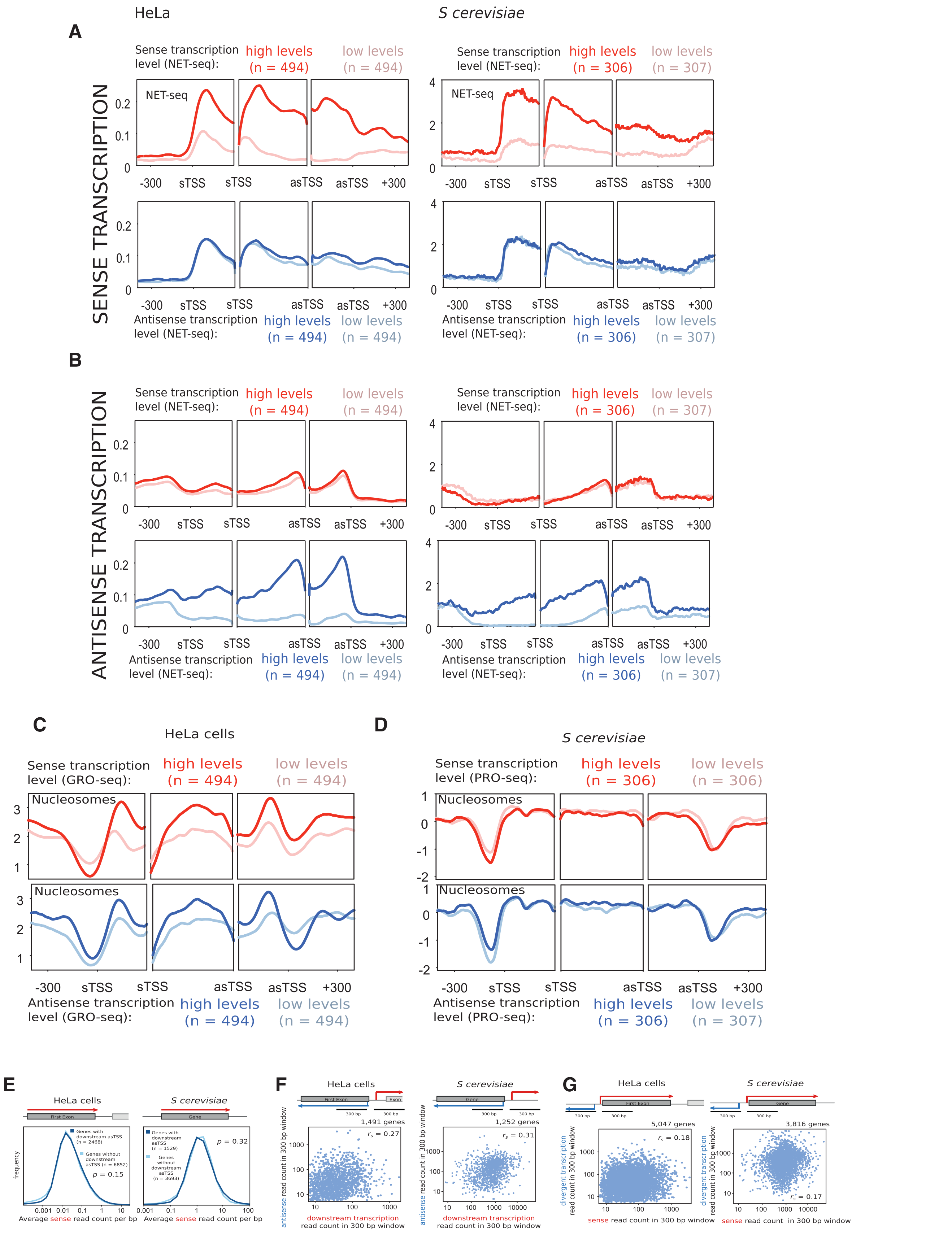
A
B
C
D
E
F
G
HeLa
S cerevisiae
SENSE TRANSCRIPTION
ANTISENSE TRANSCRIPTION
Sense transcription level (NET-seq):
high levels (n = 494)
low levels (n = 494)
high levels (n = 306)
low levels (n = 307)
NET-seq
-300 sTSS sTSS asTSS asTSS +300
Antisense transcription level (NET-seq):
HeLa cells
Sense transcription level (GRO-seq):
Sense transcription level (PRO-seq):
Nucleosomes
Antisense transcription level (GRO-seq):
Antisense transcription level (PRO-seq):
high levels (n = 306)
low levels (n = 306)
First Exon
Gene
frequency
p = 0.15
p = 0.32
Average sense read count per bp
1,491 genes
1,252 genes
5,047 genes
3,816 genes
antisense read count in 300 bp window
downstream transcription read count in 300 bp window
divergent transcription read count in 300 bp window
sense read count in 300 bp window
300 bp

Figure EV1.

Figure EV2. Antisense transcription has similar associations with chromatin modifications in both yeast and humans.

A The average levels of H3K36me3 and H3K79me3 in HeLa and *S. cerevisiae* genes. For each trio of panels, the left panel shows average levels around the sTSS, the right panel shows the average levels around the asTSS, and the middle panel shows the average level within thirty equal sized bins within the region bound by the sTSS and asTSS. Genes considered are selected from those that contained an asTSS, as defined in Fig 1. Shown in red are two sets of genes—those with high levels of sense transcription (dark red), and those with low levels (pale red), determined by GRO-seq in HeLa cells, and PRO-seq in budding yeast. Shown in blue are those genes with high levels of *antisense* transcription (dark blue), and those with low levels (pale blue), determined by GRO-seq in HeLa cells, and PRO-seq in budding yeast.

B Average levels of H3K4me3 and H3K4me1, laid out as in (A).

C Average levels of H3K9ac and H3K27ac, laid out as in (A).

 © 2018 The Authors

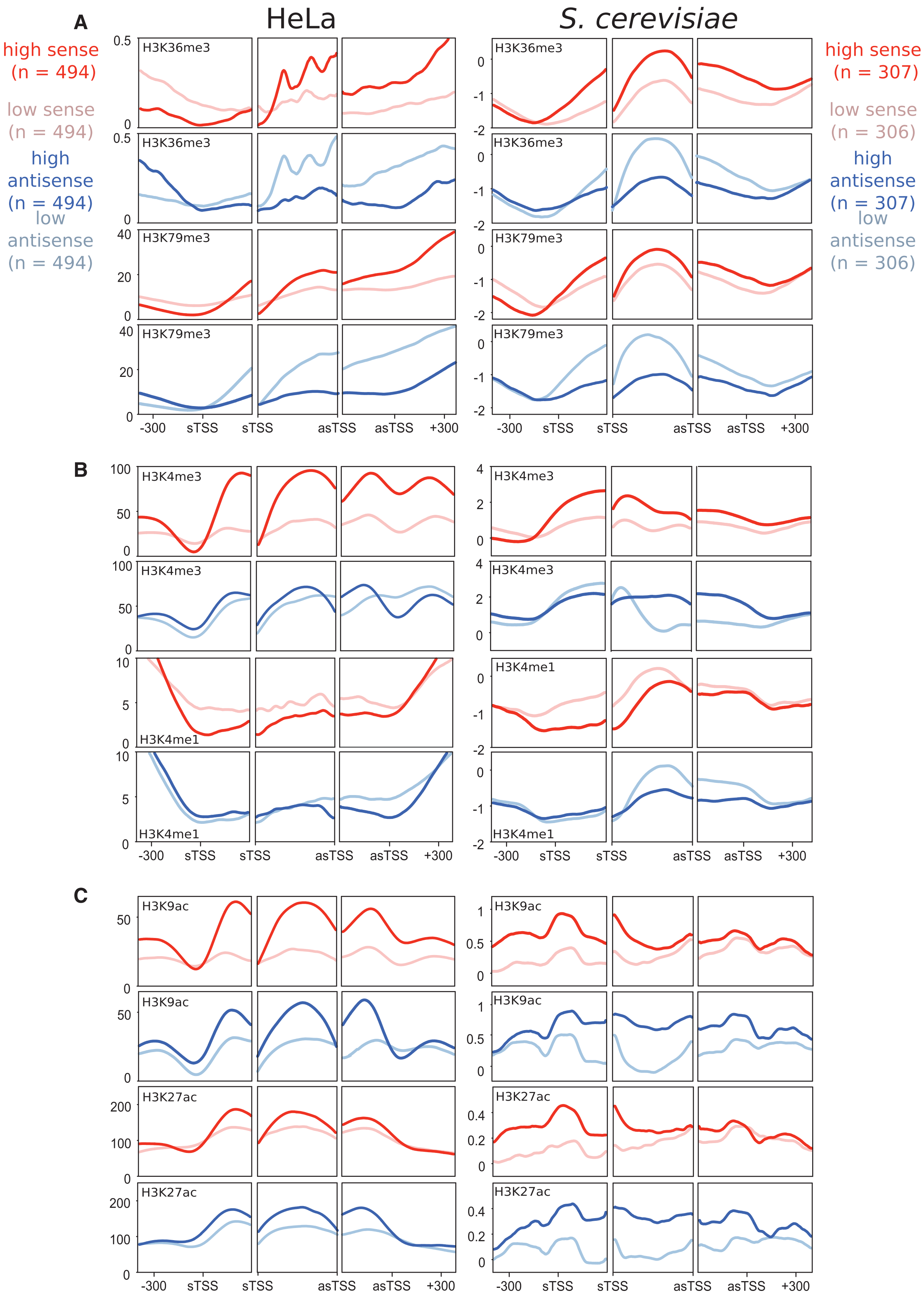
A
HeLa
S. cerevisiae
high sense (n = 494)
low sense (n = 494)
high antisense (n = 494)
low antisense (n = 494)
high sense (n = 307)
low sense (n = 306)
high antisense (n = 307)
low antisense (n = 306)
H3K36me3
H3K79me3
-300
sTSS
asTSS
+300
B
H3K4me3
H3K4me1
C
H3K9ac
H3K27ac

Figure EV2.

Figure EV3. Nuclear and cytoplasmic RNA-FISH distributions for *GAL1* foci and dynamics of transcription and transcript processing.

A The distribution of RNA foci in the nucleus or cytoplasm from WT with high or low antisense transcription, as indicated.

B Plots showing the probability density for mean production rate (left panel), nuclear processing rate (middle panel) and degradation rate (right panel) for WT strains with high or low antisense transcription, as indicated. The most likely rate is indicated above each plot.

C Estimates of transcript stability for sense transcripts from 1,529 genes with an asTSS (dark blue) or 3,693 genes without one (light blue). Left panel, ratio of sense RNA-seq over sense NET-seq as an estimate of transcript stability, middle and right panels transcript stability using data sets indicted. The frequency plots show that the majority of transcripts have higher stability when expressed from genes with an antisense transcript.

D The distribution of RNA foci in the nucleus or cytoplasm for GFP-tagged loci as indicated.

 © 2018 The Authors

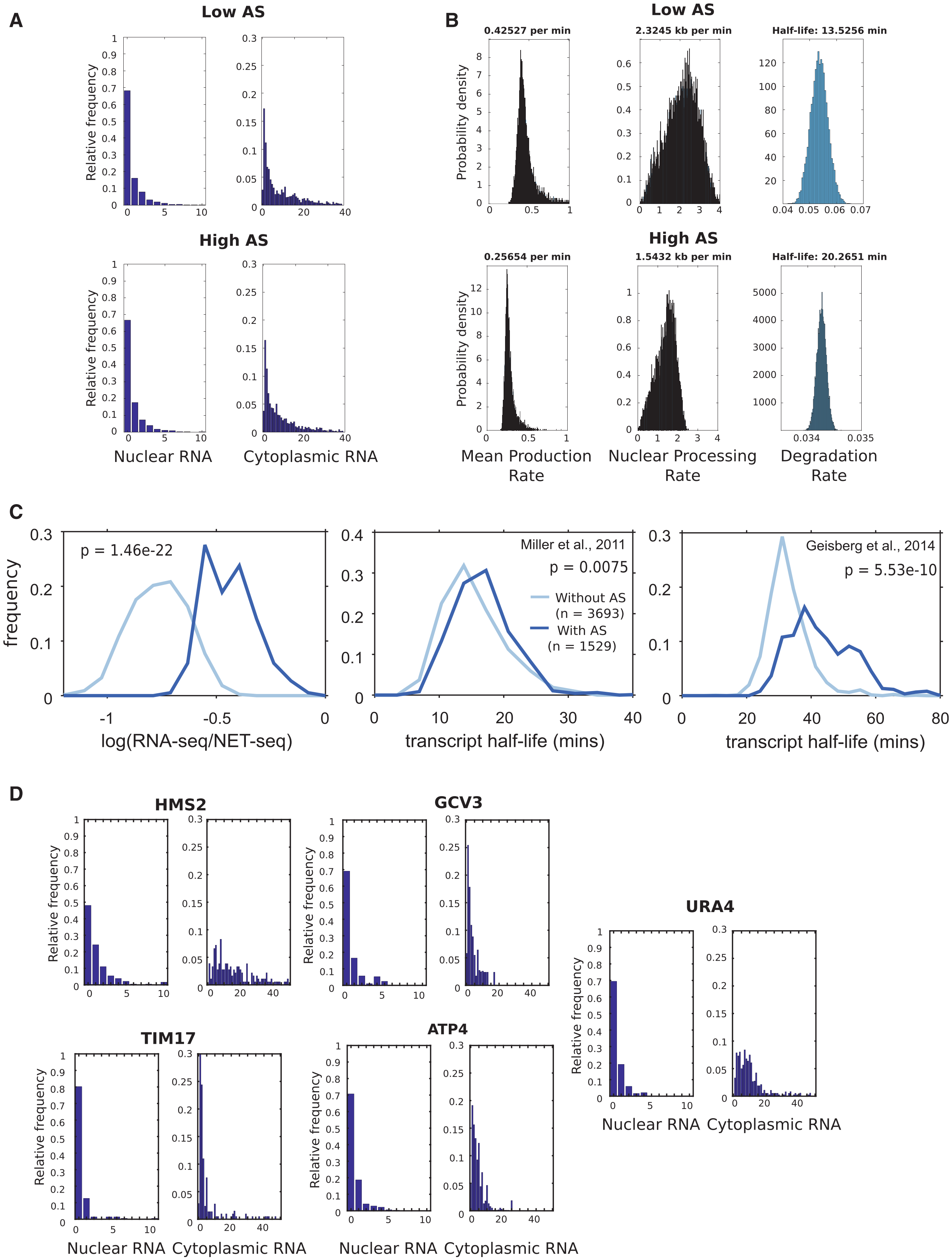
A
Low AS
High AS
Relative frequency
Nuclear RNA
Cytoplasmic RNA
B
Low AS
0.42527 per min
2.3245 kb per min
Half-life: 13.5256 min
High AS
0.25654 per min
1.5432 kb per min
Half-life: 20.2651 min
Probability density
Mean Production Rate
Nuclear Processing Rate
Degradation Rate
C
frequency
p = 1.46e-22
log(RNA-seq/NET-seq)
Miller et al., 2011
p = 0.0075
Without AS
(n = 3693)
With AS
(n = 1529)
transcript half-life (mins)
Geisberg et al., 2014
p = 5.53e-10
transcript half-life (mins)
D
HMS2
GCV3
URA4
TIM17
ATP4
Relative frequency
Nuclear RNA
Cytoplasmic RNA

Figure EV3.

Figure EV4. The presence of an antisense transcript can alter how histone acetylation levels change following deletion of a histone modifying enzyme.

A Average levels of H3K9, K14 and H4 acetylation in all budding yeast genes for both genes with and without an asTSS (top panels) or average difference in H3K9, K14 or H4 acetylation between mutant strains and wild type for both genes with and without an asTSS (bottom panels).

B Average antisense transcript levels do not change upon *SET3* deletion. Distribution of transcript levels in WT and *set3Δ* strains, obtained from Kim *et al* (2012); and transcription levels in WT and *rco1*Δ strains, obtained from Churchman and Weissman (2011), for those 1,529 antisense transcripts considered in this study. In contrast to the *rco1*Δ, there is no significant change in the genome-wide levels of these antisense transcripts, suggesting there is no overall increase or decrease in antisense transcription upon *SET3* deletion.

C H3K4me2/3 does not change upon *SET3* deletion. Levels of H3K4me2 and H3K4me3 relative to histone H3 at the engineered *GAL1* gene containing the altered *ADH1* terminator (T) as measured by ChIP-qPCR at the primer positions indicated in the schematic below in the strains with high and low antisense in the presence and absence of *SET3*. $N = 2$, error bars are SEM.

D, E Nuclear and cytoplasmic RNA-FISH distributions for *GAL1* foci and dynamics of transcription and transcript processing. (D) The distribution of foci in the nucleus or cytoplasm from *set*3Δ with high or low antisense transcription, as indicated. (E) Plots showing the probability density for mean production rate (left panel), elongation/export rate (middle panel) and degradation rate (right panel) for *set*3Δ strains with high or low antisense transcription, as indicated. The most likely rate is indicated above each plot.

© 2018 The Authors

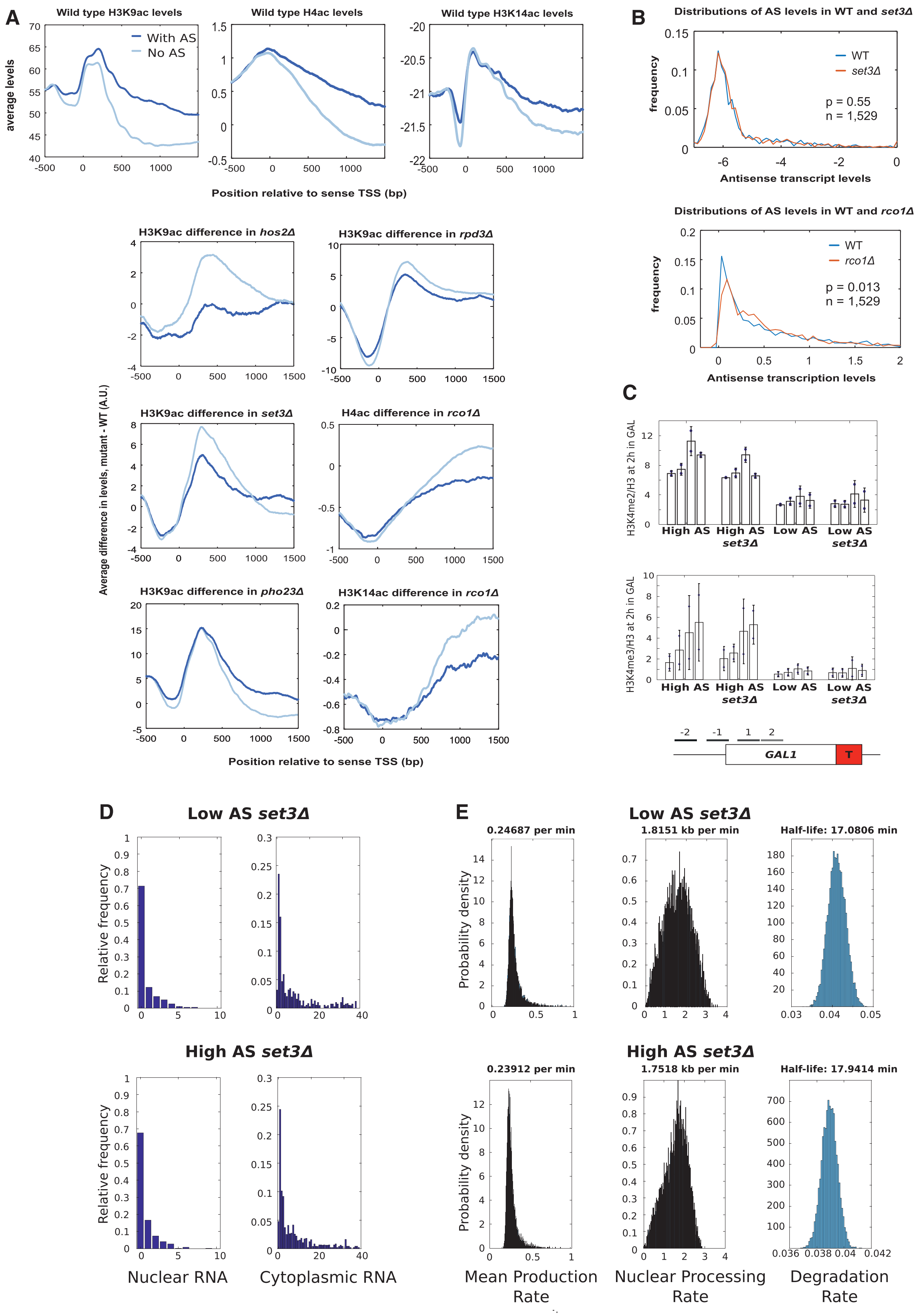
A
Wild type H3K9ac levels
Wild type H4ac levels
Wild type H3K14ac levels
With AS
No AS
average levels
Position relative to sense TSS (bp)
H3K9ac difference in hos2Δ
H3K9ac difference in rpd3Δ
H3K9ac difference in set3Δ
H4ac difference in rco1Δ
H3K9ac difference in pho23Δ
H3K14ac difference in rco1Δ
Average difference in levels, mutant - WT (A.U.)
Position relative to sense TSS (bp)
B
Distributions of AS levels in WT and set3Δ
WT
set3Δ
p = 0.55
n = 1,529
frequency
Antisense transcript levels
Distributions of AS levels in WT and rco1Δ
WT
rco1Δ
p = 0.013
n = 1,529
frequency
Antisense transcription levels
C
H3K4me2/H3 at 2h in GAL
High AS
High AS set3Δ
Low AS
Low AS set3Δ
H3K4me3/H3 at 2h in GAL
High AS
High AS set3Δ
Low AS
Low AS set3Δ
-2
-1
1
2
GAL1
T
D
Low AS set3Δ
Relative frequency
High AS set3Δ
Relative frequency
Nuclear RNA
Cytoplasmic RNA
E
Low AS set3Δ
0.24687 per min
1.8151 kb per min
Half-life: 17.0806 min
Probability density
High AS set3Δ
0.23912 per min
1.7518 kb per min
Half-life: 17.9414 min
Probability density
Mean Production Rate
Nuclear Processing Rate
Degradation Rate

Figure EV4.
